# Comparison of QSOFA and sirs scores for the prediction of adverse outcomes of secondary peritonitis among patients admitted on the adult surgical ward in a tertiary teaching hospital in Uganda: a prospective cohort study

**DOI:** 10.1186/s12873-021-00528-x

**Published:** 2021-11-06

**Authors:** Emmanuel Nkonge, Olivia Kituuka, William Ocen, Herbert Ariaka, Alfred Ogwal, Badru Ssekitoleko

**Affiliations:** 1 Department of Surgery, Villa Maria Hospital, P.O Box 32, Masaka, Uganda; 2 Department of Surgery, Makerere University, College of Health Sciences, P. O. Box 7072, Kampala, Uganda; 3 Department of Surgery, Lira University, P.O. Box 1035, Lira, Uganda; 4 Department of Surgery, Uganda Heart Institute, Kampala, Uganda; 5 Department of Surgery, Maridi County Hospital, Maridi, Western Equatoria State South Sudan

**Keywords:** Secondary peritonitis, Adverse outcomes, qSOFA, SIRS

## Abstract

**Background:**

SIRS and qSOFA are two ancillary scoring tools that have been used globally, inside and outside of ICU to predict adverse outcomes of infections such as secondary peritonitis. A tertiary teaching hospital in Uganda uses SIRS outside the ICU to identify patients with secondary peritonitis, who are at risk of adverse outcomes. However, there are associated delays in decision making given SIRS partial reliance on laboratory parameters which are often not quickly available in a resource limited emergency setting. In response to the practical limitations of SIRS, the sepsis-3 task force recommends qSOFA as a better tool. However, its performance in patients with secondary peritonitis in comparison to that of SIRS has not been evaluated in a resource limited setting of a tertiary teaching hospital in a low and middle income country like Uganda.

**Objective:**

To compare the performance of qSOFA and SIRS scores in predicting adverse outcomes of secondary peritonitis among patients on the adult surgical wards in a tertiary teaching hospital in Uganda.

**Methods:**

This was a prospective cohort study of patients with clinically confirmed secondary peritonitis, from March 2018 to January 2019 at the Accident and Emergency unit and the adult surgical wards of a tertiary teaching hospital in Uganda. QSOFA and SIRS scores were generated for each patient, with a score of ≥2 recorded as high risk, while a score of < 2 recorded as low risk for the adverse outcome respectively. After surgery, patients were followed up until discharge or death. In-hospital mortality and prolonged hospital stay were the primary and secondary adverse outcomes, respectively. Sensitivity, specificity, PPV, NPV and accuracy at 95% confidence interval were calculated for each of the scores using STATA v.13.

**Results:**

A total of 153 patients were enrolled. Of these, 151(M: F, 2.4:1) completed follow up and were analysed, 2 were excluded. Mortality rate was 11.9%. Fourty (26.5%) patients had a prolonged hospital stay. QSOFA predicted in-hospital mortality with AUROC of 0.52 versus 0.62, for SIRS. Similarly, qSOFA predicted prolonged hospital stay with AUROC of 0.54 versus 0.57, for SIRS.

**Conclusion:**

SIRS is superior to qSOFA in predicting both mortality and prolonged hospital stay among patients with secondary peritonitis. However, overall, both scores showed a poor discrimination for both adverse outcomes and therefore not ideal tools.

## Background

The use of scoring systems for surgical risk assessment in clinical practice has been known since 1941 when the American society of Anesthesiologists (ASA) developed a physical status scoring system for patients undergoing surgery. Since then, various efforts to find an ideal scoring system that correctly predicts the risk of mortality continues to occupy medical scientists [1]. Finding an ideal scoring tool is key in accurately predicting outcomes and selection of treatment options [2].

Despite the global advances in surgical practice and care, intra-abdominal infections arising from secondary peritonitis still remain one of the most significant causes of morbidity and mortality world over [3]. In the setting of septic shock, mortality of up to 30% has been reported [4]. In order to accurately predict these adverse outcomes of secondary peritonitis, a number of risk assessment scoring tools have been developed and used with various performance levels in different clinical settings. SIRS and qSOFA are two such ancillary scoring tools that have been used extensively inside and outside the Intensive Care Unit (ICU) setting globally. QSOFA is a surrogate for SOFA and it assigns one point for each of its 3 parameters which are systolic blood pressure less than 100 mmHg, respiratory rate greater or equal to 22 breaths per minute and Glasgow coma scale of less than 15. According to the third international consensus definitions for sepsis and septic shock which all can be a sequelae of secondary peritonitis, a SOFA score of ≥2 is associated with mortality in excess of 10% [5]. This was the basis for stratifying the qSOFA which is used for bedside assessment of patients with infection who are at risk of adverse outcomes outside ICU into 2 groups; ≥ 2 was high risk and < 2 was low risk. SIRS scores were similarly stratified so that the two scores could easily be compared. SIRS is based on 4 physiological variables (respiratory rate of greater than 20 breaths per minute, body temperature of greater than 38 or less than 34 degrees Celsius, heart rate of greater than 90 beats per minute and white blood cell count of greater than 12,000per cubic millimeters or less than 4000 per cubic millimeters) for which it assigns a point to each [6]. A SIRS score of ≥2 was considered high risk and a score of < 2 as low risk.

In response to practical limitations of SIRS, the third international consensus conference of 2016 on sepsis (sepsis-3) introduced and recommended qSOFA as a better tool than SIRS for predicting adverse outcomes among patients with infections outside the ICU [5]. Mulago hospital which is a tertiary referral hospital in a low and middle income country, uses SIRS outside ICU to assess disease severity and identify patients at risk of sepsis and other adverse outcomes arising from secondary peritonitis. However, SIRS relies on laboratory parameters which have a long turnaround time in some hospitals in low and middle income countries and therefore result in delays of scoring patients and ultimately in decision making in an emergency setting. These drawbacks have led to the need for a simpler, accurate bedside clinical tool which does not rely on laboratory parameters and can be used by clinicians at centers which experience delays in getting laboratory results for surgical emergencies. Clinicians would then be able to identify patients who are at high risk of adverse outcomes easily and earlier and prepare for the appropriate care to avert the potential adverse outcomes in the pre-operative period as well as provide early post operative ICU care where needed and thus probably improve the outcomes of patients with secondary peritonitis.

Since its inception, qSOFA has been validated in many centers and found to be a better predictor of adverse outcomes in patients with infections outside the ICU compared to SIRS [7, 8]. We needed to assess whether these findings were applicable in the setting of a tertiary teaching hospital in a low and middle income country like Uganda. This study aimed to find out whether the performance of qSOFA is superior to that of SIRS in predicting adverse outcomes of secondary peritonitis among patients admitted on the adult surgical wards in Mulago hospital, Uganda.

## Methods

This was a prospective cohort study conducted between March 2018 and January 2019. The aim of this study was to determine whether qSOFA was superior to SIRS in predicting adverse outcomes of peritonitis among patients 13 years and above at Mulago National Referral Hospital. Mulago National Referral Hospital is a 1500 bed tertiary teaching hospital located in Kampala which is the capital city of Uganda. This study was based in the Accident and Emergency (A&E) department which handles surgical emergencies like secondary peritonitis, among others. Here patients are resuscitated and stabilized before definitive surgical interventions are undertaken. Emergency surgical operations are carried out in the A & E theatre with an average of two laparotomies due to secondary peritonitis being performed daily. After surgery, the patients are temporarily admitted to the Emergency ward before transfer to their definitive general surgical wards. Patients who are 13 years and above are admitted on the adult surgical unit while those 12 years and below are admitted on the paediatric surgical ward. This study considered patients with secondary peritonitis who would be admitted on the adult surgical wards after emergency treatment from the Accident and Emergency Unit. Patients who were younger than 13 years and were subsequently admitted on the paediatric surgical wards were excluded from the study.

### Sampling

The study population was all patients who presented with secondary peritonitis at the Accident and Emergency Unit. Inclusion criteria was all patients 13 years and above who presented with secondary peritonitis at the Accident and emergency unit and were later admitted on the adult surgical wards. Patients who did not complete the follow up period were excluded from the study. We sampled 153 patients diagnosed and admitted with secondary peritonitis in the A & E department of Mulago Hospital between March 2018 and January 2019 These patients were followed up on the adult surgical wards until discharge from hospital or death. We excluded 2 patients who were lost to follow up from the analysis and therefore analysed data from 151 patients.

### Data analysis

Descriptive statistics for the patients were summarized using proportions for categorical variables, whereas continuous and discrete variables were summarized as means (standard deviation), or median (Interquartile Range (IQR)) depending on the distribution of data. Level of significance was taken to be 5%.

The proportions of patients with secondary peritonitis were scored as high risk(a score greater or equal to two) or low risk(a score of less than two) using the qSOFA or SIRS scores. Patients falling in either category of high or low risk were recorded in dichotomous 2 × 2 contingency table against presence or absence of an adverse outcome. Adverse outcomes were prolonged hospital stay of more than 7 days and death. The sensitivity, specificity, positive predictive value, negative predictive value and accuracy and their 95% confidence intervals (CI) were computed. True positives were those who scored high risk and actually got an adverse outcome, false positives were those who did not get an adverse outcome but were scored high risk, false negatives got the adverse outcome but were scored low risk and true negatives were those who were scored low risk and did not get the adverse outcome. Since none of the two scoring tools is the gold standard, the truth against which they were compared was determined clinically.

Accuracy was obtained by constructing the receiver characteristic operating curve. The tool with a wider area under the curve was considered to be superior to the other.

All the above parameters were compared for both qSOFA and SIRS, and the better performing tool of the two reported accordingly.

## Results

### Patients’ demographic characteristics

There were 106(70.2%) males and 45(29.8%) females, giving a M:F of 2.4:1. Majority of the patients (55%),were between 13 and 29 years, Table 1.

**Table 1:** Patients’ demographic characteristics

Variable	***n*** = 151	%
**Age (years)**		
13–29	83	55.0
30–59	48	31.8
> 60	20	13.2
**Sex**		
Male	106	70.2
Female	45	29.8

### Adverse outcomes and distribution of qSOFA and SIRS scores

At the end of follow up, 18 patients were dead, giving an overall mortality of 11.9% for secondary peritonitis. Majority of the deaths (66.7%) occurred in the elderly patients (older than 50 years). Forty (26.5%) patients had a prolonged length of stay (Table 2).

**Table 2:** Adverse outcomes and distribution of the scores

Score	Died	%	Alive	%	LOS > 7	%	LOS ≤ 7	%
	*n* = 18		*n* = 133		*n* = 40		*n* = 111	
**qSOFA ≥ 2**	2	11.1	16	12.0	7	17.5	11	9.9
**qSOFA < 2**	16	88.9	117	88.0	33	82.5	100	90.1
**SIRS ≥ 2**	15	83.3	80	60.2	29	72.5	66	59.5
**SIRS < 2**	3	16.7	53	39.8	11	27.5	45	40.5

### Sensitivity and specificity

QSOFA predicted mortality with a sensitivity of 11.1% (95%CI,1.38–34.71) and specificity of 88.0%(95%CI,81.20–92.96), compared to 88.3% (95%CI,58.58–96.42) and 40.6% (95%CI,32.18–49.46), respectively for SIRS.

Conversely, qSOFA predicted prolonged length of hospital stay with a sensitivity of 17.5%(95%CI,7.34–32.78) and specificity of 90.1%(95%CI,82.96–94.95), compared to 72.5%(95%CI,56.11–85.40) and 41.4%(95%CI,32.1–51.18), respectively for SIRS.

QSOFA was a more specific but less sensitive tool, while SIRS was a far more sensitive but less specific tool for the prediction of both mortality and prolonged hospital stay as a result of having secondary peritonitis outside the ICU setting in Mulago hospital (Table 3).

**Table 3:** Sensitivity and Specificity of qSOFA and SIRS in predicting mortality and prolonged hospital stay

Score		Mortality			LOS > 7	
	Se(%)	95%CI	Sp(%)	95%CI	Se(%) 95%CI	Sp(%) 95%CI
**qSOFA**	11.1	1.38–34.71	88.0	81.20–92.96	17.5 7.34–32.78	90.1 82.96–94.95
**SIRS**	88.3	58.58–96.42	40.6	32.18–49.46	72.5 56.11–85.40	41.4 32.1–51.18

### Positive and negative predictive values

QSOFA predicted mortality with a PPV of 11.1% (95%CI,1.38–34.71) and NPV of 88% (95%CI,81.20–92.96) compared to 16.0% (95%CI,9.22–24.95) and 94.7% (95%CI,85.38–98.90) respectively for SIRS.

Conversely, qSOFA predicted prolonged hospital stay with a PPV of 38.9%(95%CI,17.30–64.25) and NPV of 75.2% (95%CI,66.96–82.26), compared to 30.9% (95%CI,21.73–41.22) and 80.7% (95%CI,68.09–89.95) respectively for SIRS.

SIRS had a superior predictive value, in comparison to qSOFA, for both mortality and prolonged hospital stay resulting from secondary peritonitis (Table 4).

**Table 4:** Positive and Negative predictive values of qSOFA and SIRS for mortality and prolonged hospital stay

Score		Mortality				LOS > 7		
	PPV(%)	95%CI	NPV(%)	95%CI	PPV(%)	95%CI	NPV(%)	95%CI
**qSOFA**	11.1	1.38–34.71	88.0	81.20–92.96	38.9	17.30–64.25	75.2	66.96–82.26
**SIRS**	16.0	9.22–24.95	94.7	85.38–98.90	30.9	21.73–41.22	80.7	68.09–89.95

### Accuracy

**Fig. 1:**
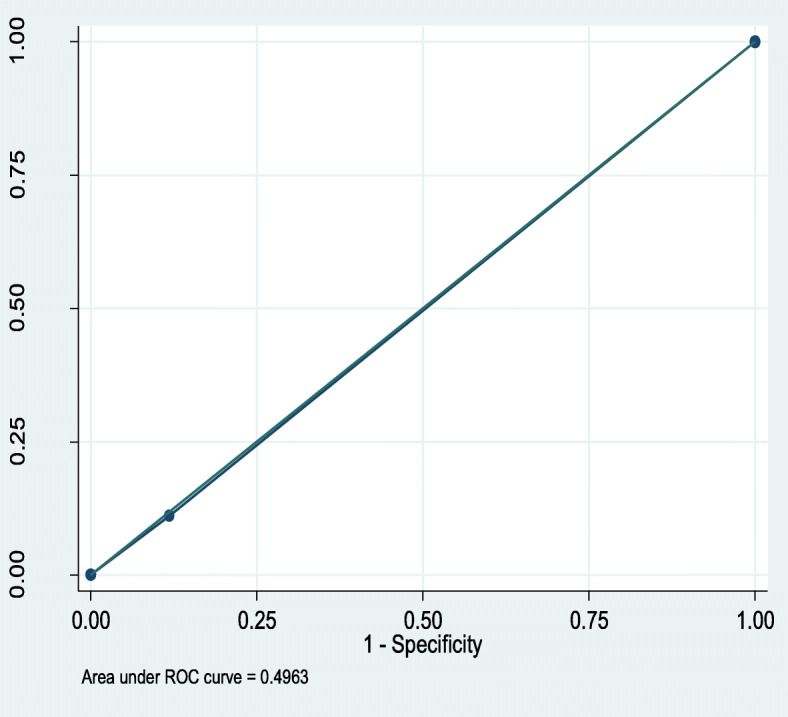
Receiver operating characteristic (ROC) curve for prediction of mortality by qSOFA

**Fig. 2:**
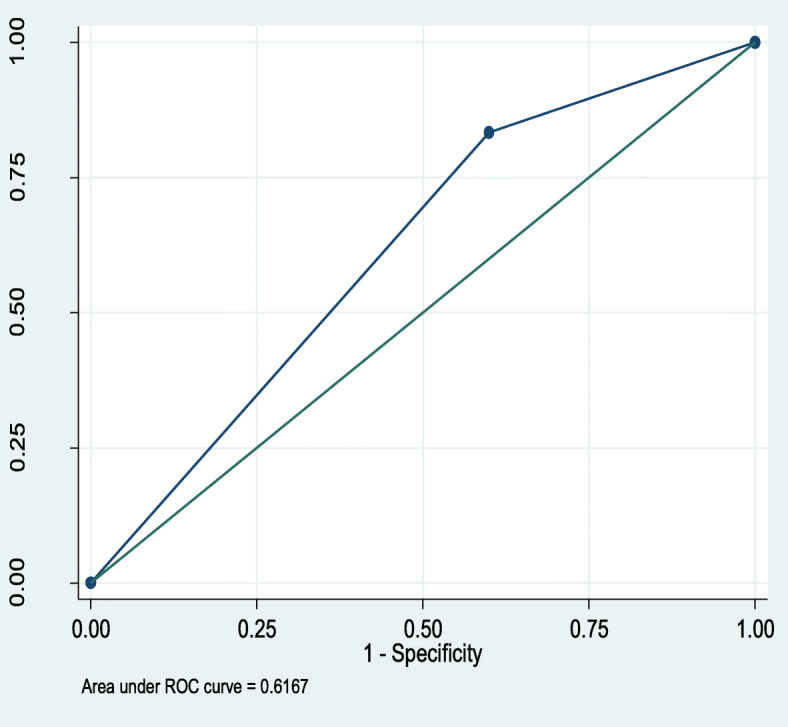
ROC curve for prediction of mortality by SIRS. Conversely, qSOFA predicted prolonged hospital stay with an AUROC of 0.54(95%CI,0.45–0.62), compared to 0.57(95%CI,0.49–0.65),for SIRS, Figs. 3 and 4 respectively

QSOFA predicted mortality with an Area under receiver operating characteristic curve (AUROC) of 0.50 (95%CI,0.42–0.58), compared to 0.62 (95%CI,0.54–0.70) for SIRS, Figs. 1 and 2 respectively.

**Fig. 3:**
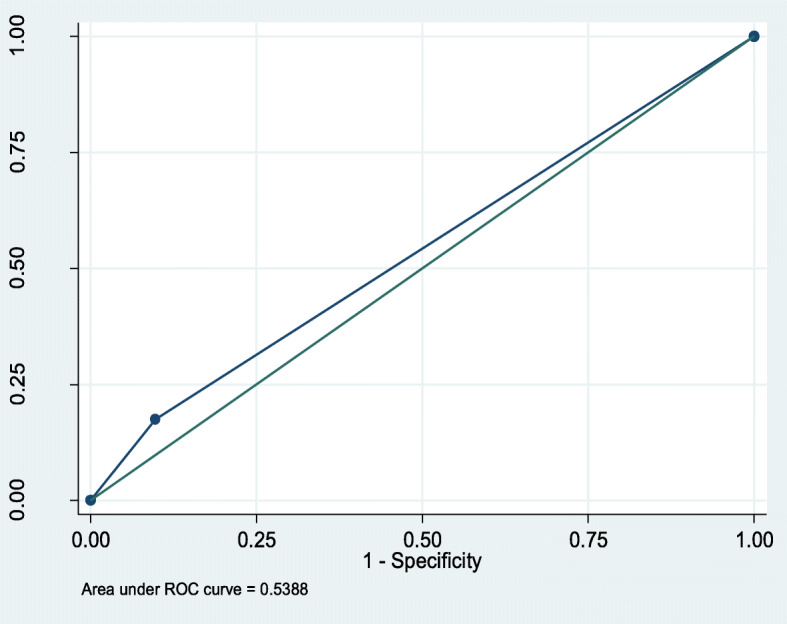
ROC curve for prediction of prolonged hospital stay by qSOFA

**Fig. 4:**
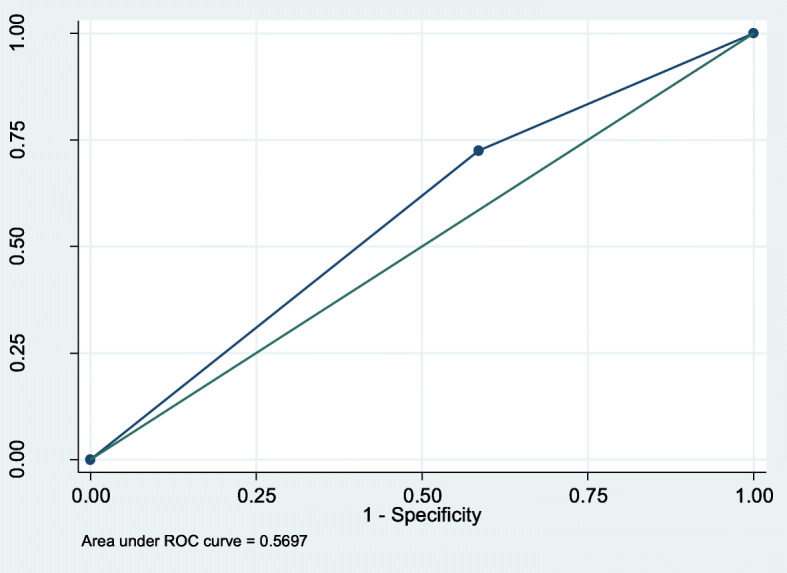
ROC curve for prediction of prolonged hospital stay by SIRS

Overall, SIRS predicted both mortality and prolonged hospital stay, resulting from secondary peritonitis outside the ICU setting in Mulago hospital more accurately than qSOFA.

## Discussion

The third international consensus on sepsis 2016, introduced qSOFA as a better tool than SIRS, for identifying patients with infections, at risk of adverse outcomes. We set out to compare the performance of qSOFA and SIRS scores for the prediction of in-hospital mortality as a primary adverse outcome, and prolonged length of hospital stay as a secondary adverse outcome, among patients with secondary peritonitis at Mulago National referral and teaching hospital. Majority of the patients in this study were young. This finding is consistent with what Wabwire and Saidi reported in their study on stratified evaluation of secondary peritonitis [9]. There were more males compared to females in this study. A similar observation has been reported by previous studies [3, 9–15].

In this study SIRS predicted mortality and prolonged hospital stay more accurately than qSOFA. These findings were similar to those in a retrospective study of 1045 patients who presented in the emergency department with infection where SIRS showed a better performance for predicting infection than qSOFA [16]. The patients in that study were non surgical patients while our study recruited patients prospectively who eventually underwent emergency surgery for secondary peritonitis. However in another study which assessed QSOFA, SOFA and SIRS scores’ accuracy at predicting infection and mortality among surgical intermediate and ICU patients, it was found that none of the scores was sufficiently able to predict suspected infection in these patients [17]. Our study stratified SIRS scores and qSOFA scores into high risk and low risk groups which could have included more low risk patients in the high risk group for SIRS. In so doing this could have resulted in higher assessment of infections and mortality than the other study.

Although the overall mortality rate in this study was comparable to what has been reported by other studies [9, 15, 18] is still quite high and underscores the need for early identification of at risk patients for prompt intervention. The higher mortality rate among elderly patients could be probably because the elderly are likely to have poor physiological reserves and or comorbidities and possibly late diagnosis and also late patient presentation. In this study, prolonged hospital stay was attributed to the attendant complications of secondary peritonitis, including but not limited to surgical site infections, burst abdomen, and relaparotomy.

A meta-analysis of 8 studies to compare qSOFA and SIRS in mortality of patients in the Emergency Department with infections showed that scores > 2 for both scores were strongly associated with mortality. QSOFA > 2 was more specific in predicting mortality while SIRS > 2 was more sensitive [19]. A prospective multi-centre clinical trial demonstrated that qSOFA was modestly better in accurately predicting mortality but was less sensitive for in-hospital mortality among patients with suspected infection in the emergency department [20]. In this study, qSOFA was also more specific, but less sensitive tool, while SIRS was a more sensitive, but less specific tool, for the prediction of both mortality and prolonged hospital stay. Findings akin to these, have been reported by Singer et al. in their sepsis 3 report [5], and subsequent studies by Finkelsztein et al., Freund et al. and Churpek et al. [7, 8, 21]. SIRS had a superior predictive value for both mortality and prolonged length of hospital stay, compared to qSOFA. This finding contrasts with that from previous studies [8, 22, 23]. SIRS was superior to qSOFA in predicting both mortality and prolonged hospital stay, in this study, consistent with the findings of Askim et al. [22].

The above findings however, contrast with those reported by several studies that found qSOFA to be superior to SIRS in predicting in-hospital mortality and ICU admission [7, 8, 21, 24]. This disparity could be because a few notable differences between the aforementioned studies and this study. Churpek and Finkelsztein included patients being transferred from wards to the ICU, since they were assessing prediction of ICU stay by both scores as a secondary outcome [7, 21]. Such patients are more likely to have high qSOFA scores since they are critically ill, with multiple organ dysfunction, compared to stable patients in the A&E. None of the patients in this study was transferred to ICU. Freund et al. calculated qSOFA scores by collecting its parameters at their worst level during the patients’ entire hospital stay, that is, the highest respiratory rate, lowest systolic blood pressure, and lowest Glasgow coma scale. It is not clear from their study though, whether the same was applied to the SIRS scores. This could have biased their results [8]. In our study, both qSOFA and SIRS scores were calculated at admission only, during the patients’ entire hospital stay, irrespective of whether there was or there was no change in the parameters, from which they are generated.

### Limitations of the study

In this study, Clinical and laboratory parameters used to generate both qSOFA and SIRS scores, were collected at admission only, during the patients’ entire hospital stay. Cognizant of the fact that, these keep changing from time to time especially when patients deteriorate, could have resulted in many patients not meeting the criteria for both scores and yet developed the adverse outcomes (High false negative rate). We further acknowledge that the cause of secondary peritonitis could also have contributed to the outcomes of the patients.

Both qSOFA and SIRS scores do not consider parameters like patients’ age, sex, and presence of comorbidities, all of which have the potential to modify the outcomes of interest in this study. However, since baseline risk associated with those parameters was considered during the development of qSOFA score, this may not have affected the results significantly.

## Conclusions

SIRS score is superior to qSOFA score in predicting both in-hospital mortality and prolonged hospital stay of patients with secondary peritonitis in Mulago hospital. Overall however, both qSOFA and SIRS scores showed poor discrimination of both adverse outcomes in this study and therefore not ideal for this purpose.

SIRS score is more sensitive but less specific tool, while qSOFA is a more specific but less sensitive tool for predicting both in-hospital mortality and prolonged hospital stay of patients with secondary peritonitis in the surgical emergency and adult surgical wards.

## Data Availability

The datasets used or analyzed during this study are not publicly available because this data was for academic research but are available from the primary author Dr. Emmanuel Nkonge on a reasonable request.

## Abbreviations

ASA: American Society of Anesthesiologists
SOFA: Sequential organ failure assessment
qSOFA: Quick Sequential (Sepsis-related) Organ Failure Assessment
SIRS: Systemic Inflammatory Response Syndrome
ICU: Intensive care unit
APACHE: Acute Physiology and Chronic Health Evaluation
ACCP: American College of chest physicians
SCCM: Society of critical care medicine
ESICM: European society of intensive care medicine
Se: Sensitivity
Sp: Specificity
PPV: Positive predictive value
NPV: Negative predictive value
AUROC: Area under receiver operating characteristic curve
LOS: Length of hospital stay
